# Dynamic and static biomechanical traits of cardiac fibrosis

**DOI:** 10.3389/fbioe.2022.1042030

**Published:** 2022-10-31

**Authors:** Han Liu, Pengbei Fan, Fanli Jin, Guoyou Huang, Xiaogang Guo, Feng Xu

**Affiliations:** ^1^ Henan Key Laboratory of Chinese Medicine for Respiratory Disease, Academy of Chinese Medical Sciences, Henan University of Chinese Medicine, Zhengzhou, China; ^2^ Collaborative Innovation Center for Chinese Medicine and Respiratory Diseases Co-Constructed by Henan Province and Education Ministry of China, Zhengzhou, China; ^3^ Department of Engineering Mechanics, School of Civil Engineering, Wuhan University, Wuhan, China; ^4^ The First Affiliated Hospital, College of Medicine, Zhejiang University, Hangzhou, China; ^5^ The Key Laboratory of Biomedical Information Engineering of Ministry of Education, School of Life Science and Technology, Xi’an Jiaotong University, Xi’an, China; ^6^ Bioinspired Engineering and Biomechanics Center (BEBC), Xi’an Jiaotong University, Xi’an, China

**Keywords:** cardiac fibrosis, biomechanical traits, myofibroblast, mechanotransduction, mechanical model *in vitro*

## Abstract

Cardiac fibrosis is a common pathology in cardiovascular diseases which are reported as the leading cause of death globally. In recent decades, accumulating evidence has shown that the biomechanical traits of fibrosis play important roles in cardiac fibrosis initiation, progression and treatment. In this review, we summarize the four main distinct biomechanical traits (i.e., stretch, fluid shear stress, ECM microarchitecture, and ECM stiffness) and categorize them into two different types (i.e., static and dynamic), mainly consulting the unique characteristic of the heart. Moreover, we also provide a comprehensive overview of the effect of different biomechanical traits on cardiac fibrosis, their transduction mechanisms, and *in-vitro* engineered models targeting biomechanical traits that will aid the identification and prediction of mechano-based therapeutic targets to ameliorate cardiac fibrosis.

## 1 Introduction

Cardiac fibrosis, also known as myocardial fibrosis, is a common pathology in cardiovascular disease, whose mortality has been regarded as the leading cause of death globally and attracted considerable attention (Schafer et al., 2017; Mensah et al., 2019; Diseases and Injuries, 2020; Alexanian et al., 2021). Cardiac fibrosis is a relatively complex pathological process, caused by persistent or repeated exacerbations of myocardial ischemia and hypoxia (Zhao et al., 2022a), characterized by the excessive accumulation of the extracellular matrix (ECM) components (e.g., collagen and fibronectin) (Henderson et al., 2020; Frangogiannis, 2021). Although cardiac fibrosis is generally considered as a disease of the phenotypic transformation of cardiac fibroblasts and has been studied mainly from a biological perspective (Ma et al., 2018), accumulating evidence indicates that the mechanical properties of cardiac tissues, from the macroscale to the microscale, underlie cellular behavior and tissue functions (Orre et al., 2019; Guimaraes et al., 2020). In addition, biomechanical properties of tissue play a critical role in maintaining organ structure and function as well (Peyronnet et al., 2016; Wagh et al., 2021). For example, related to the diastolic function impairment and heart failure, diffuse thickening (a crucial fibrotic manifestation in the process of hypertrophic cardiomyopathy) of tissue caused by cardiac fibrosis might limit cardiac myocytes contractility and impaired ventricular function (de Jong et al., 2011; Cowling et al., 2019). Furthermore, biomechanical cues have been reported as a coconspirator of biological traits in tissue fibrosis initiation, progression and treatment response (Hadjicharalambous et al., 2021; He et al., 2021). However, due to the physical differences in each organ, different organs [e.g., heart (Lemaitre et al., 2021), liver (Kong et al., 2021), and lung (Freeberg et al., 2021)] have their specific mechanical traits as well.

In the heart, the abnormal static biomechanical traits like stiffness or microarchitecture facilitate the progress of cardiac fibrosis *in vivo* (Islam et al., 2021). The change of collagen microarchitecture can regulate myofibroblast differentiation and fibrosis independent of collagen quantity and bulk stiffness by locally modulating cellular mechanosignaling (Seo et al., 2020a). For example, changes in matrix structure and components can alter the cell-matrix and cell-cell interactions and related signal transduction (Ashworth et al., 2020; Yamada et al., 2022). The ECM microarchitecture could be altered by the contractile activity of myofibroblast containing stress fibers as well (Seo et al., 2020b; Davidson et al., 2020). Moreover, increased stiffness can activate signaling pathways that promote fibroblast proliferation and differentiation, which in turn accelerates the progression of cardiac fibrosis (Gourdie et al., 2016; Villar et al., 2022). Besides the static biomechanical traits, cardiac cells are constantly regulated by dynamic biomechanical traits, such as fluid shear and cyclic stretch as generated by blood flow and heartbeats (Fukui et al., 2021; Kotini et al., 2022). For example, abnormal mechanical stretching leads to excess proliferation and differentiation of fibroblasts, resulting in irreversible cardiac fibrosis (Yong et al., 2015; Morley et al., 2018). Dynamic overstretching can also cause microstructural remodeling of the myocardium, which is closely associated with systolic and/or diastolic dysfunction (Caporizzo and Prosser, 2022). Thus, a rigorous description of the biomechanical traits of cardiac fibrosis will contribute to a better understanding of the complex mechanism of fibrosis and the exploration of effective anti-fibrosis therapies.

**FIGURE 1:**
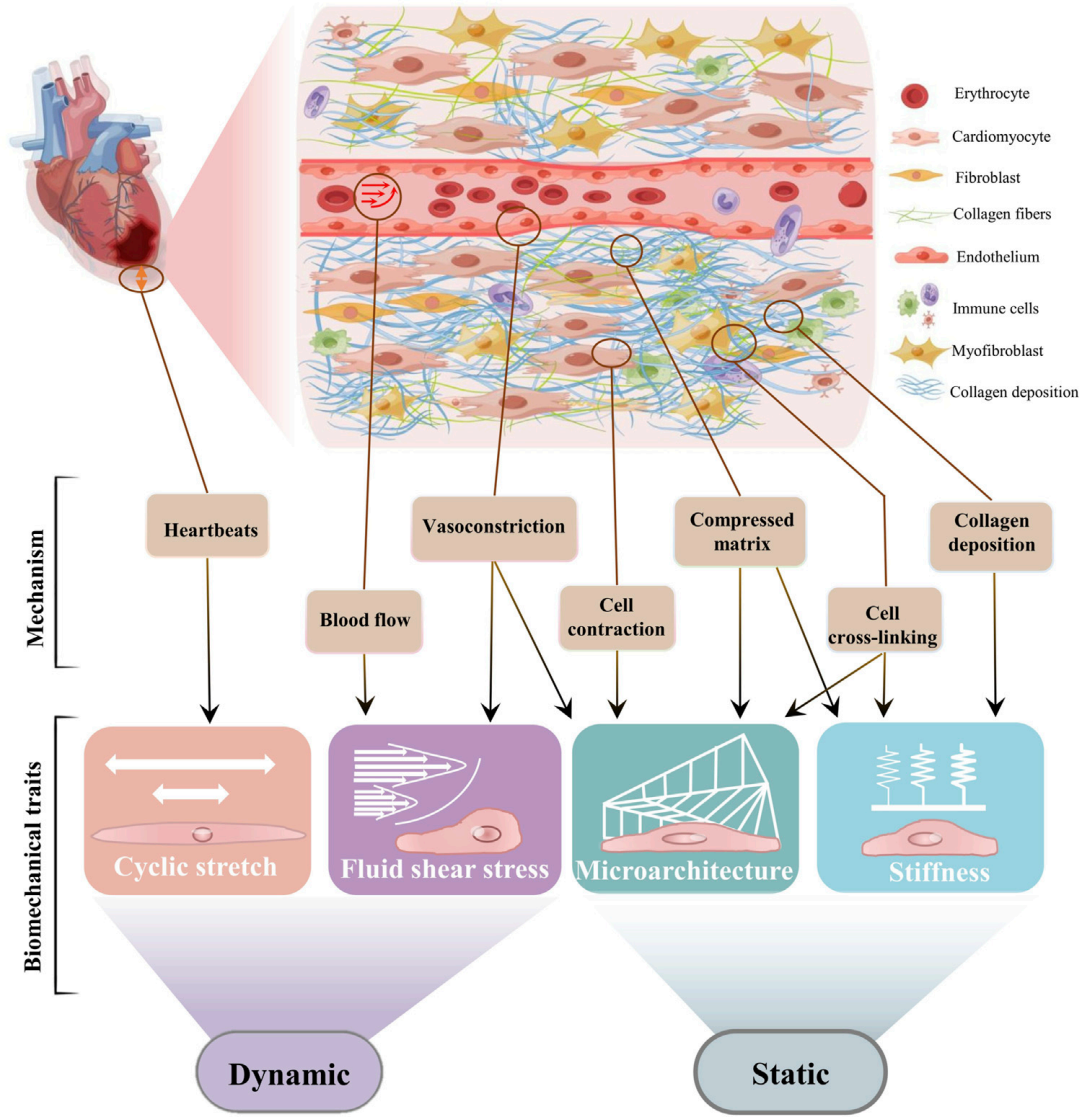
The biomechanical traits of cardiac fibrosis. Schematic diagram of cardiac fibrosis ECM was drew online using Figdraw. According to recent progresses of biomechanics, we recommend respectively from two biomechanical types to understand cardiac fibrosis. The fibrotic area must be subjected to a stretch force because of the beating of the heart. The microarchitecture and the fluid shear stress are the mechanical stress contained and transmitted by the solid phase and the liquid phase, respectively. Stiffness is defined as the ability of a material to resist deformation under external forces. The physical interaction between cardiac cells and ECM produces the physical features of cardiac fibrosis through different and interrelated mechanisms. The abnormal contraction and relaxation of the heart cause vascular stretch to increase blood fluid pressure within the site of fibrosis. Cell differentiation, increased fluid shear stress, and matrix deposition result in compressive microarchitecture. Cardiac stretch, matrix deposition, and cross-linking can respectively lead to increased stiffness at the fibrotic site. The microarchitecture leads to the stretching and alignment of the matrix, and tissue stiffening increases the differentiation of cardiac fibroblasts. Fluid flow and excessive strain activate fibroblasts, which then contribute to increased cardiac wall stress and stiffness values and changes in ECM structure.

Although there exist a few reviews on cardiac fibrosis from the aspect of cell biological mechanisms, molecular pathways and therapeutic opportunities (Frangogiannis, 2019a; Park et al., 2019; Frangogiannis, 2021), there is still a lack of one focusing on the biomechanical properties of cardiac fibrosis. Here, we summarize four distinct biomechanical traits (i.e., stretch, fluid shear stress, ECM microarchitecture, and ECM stiffness) and categorize them into two different types (i.e., dynamic and static) (Figure 1). Next, we provide a comprehensive overview of the effect of different biomechanical traits on cardiac fibrosis, their transduction mechanisms, and the *in vitro* engineering models targeting biomechanical traits. We finally conclude with a perspective on important open challenges of the role of other biophysical cues, and future directions like identification of the mechano-based therapeutic targets to ameliorate cardiac fibrosis progression.

## 2 Static biomechanical traits

### 2.1 Common static biomechanical traits during cardiac fibrosis

#### 2.1.1 Increased stiffness

Biomechanical microenvironments are associated with heart attacks and may be the source of abnormal signals that drive cardiac cells to adapt to adverse changes. As a static biomechanical characteristic, stiffness refers to the ability of tissue structure to resist elastic deformation when subjected to a force (Levine et al., 2021). Increased stiffness of myocardial tissue (∼55 kPa) is an important feature of cardiac fibrosis, due to the excessive transformation of cardiac fibroblasts and accumulation of various components of the ECM, which can be three or four times stiffer than healthy myocardium (∼10 kPa) (Berry et al., 2006). In addition, cells can also generate traction to recruit the matrix to make the fibrotic ECM denser, resulting in a significant increase in the local stiffness.

**FIGURE 2:**
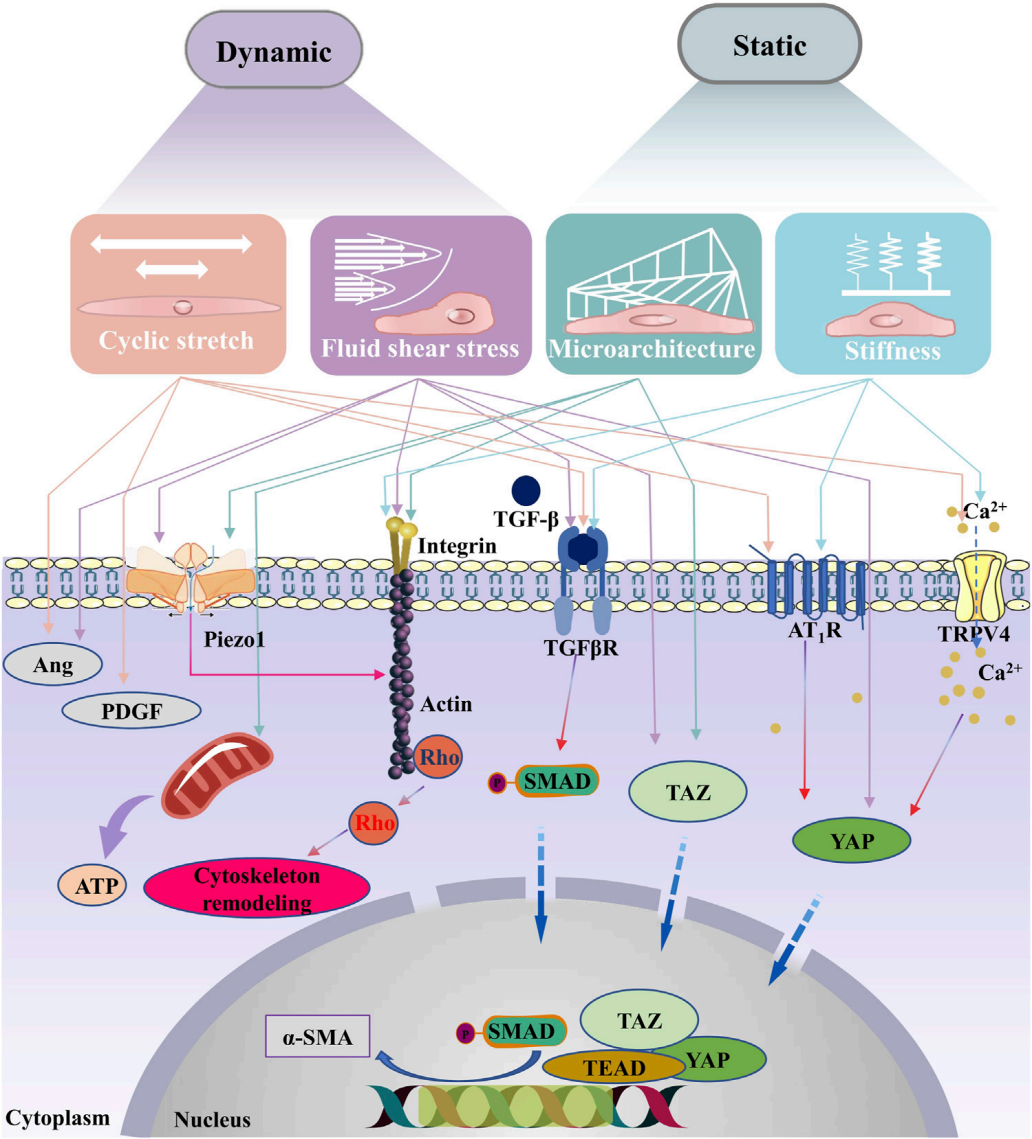
Signaling pathways associated with the biomechanical traits of cardiac fibrosis. With the changes of ECM mechanical properties during the process of cardiac fibrosis, myofibroblasts are activated . In classical signaling pathway, the TGF-β receptor’s activation induces phosphorylation of the C-terminus of SMAD. The phosphorylated SMADs then form a complex with the co-mediator SMAD, SMAD4, the complex is translocated into the nucleus, where it binds to the gene promoter. Upon myofibroblasts are activated, TGF-β is released from binding proteins in the ECM, leading to sustained activation and contraction of myofibroblasts, finally causing a vicious cycle of fibrotic progression. In addition, integrins which sense changes in external forces can also contribute to the remodeling of the cytoskeleton. Studies have shown that the activation of cell membrane surface mechanosensitive receptors (such as Piezo1, AT_1_R and TRPV4) are also key pathways in the vicious cycle of fibrosis. Correlational studies have shown that Piezo1 can be activated by shear stress, stretching and matrix microarchitecture. Ang, Angiopoietin; TGF-β, transforming growth factor-beta; PDGF, plateau-derived growth factor; α-SMA, Piezo1, piezo type mechanosensitive ion channel component 1; alpha-smooth muscle actin; TGFβR, transforming growth factor-beta receptor; TRPV4, transient receptor potential vanilloid type 4; YAP, YES-related proteins; TAZ, transcriptional coactivator with PDZ-binding motif; AT1R, angiotensin type 1 receptor; TEAD, TEA domain transcription factors; SMAD, drosophila mothers against decapentaplegic.

Although the stiffness of fibrotic myocardium (∼55 kPa) (Huyer et al., 2015) exceeds the diastolic stiffness of healthy myocardium (∼8–10 kPa) (Wang et al., 2019), it is lower than that of systolic myocardium (>100 kPa) (Huyer et al., 2015). When the myocardium is partially stiffened during cardiac fibrosis, immune cells will be recruited and activated to abnormal locations, and then profibrotic factors (e.g., cytokines, growth factors, and chemokines) will be released (Halade and Lee, 2022). Subsequently, the release of these factors [e.g., transforming growth factor-beta (TGF-β), plateau-derived growth factor (PDGF)] leads to the conversion of cardiac fibroblasts into activated myofibroblasts, promoting collagen deposition (Zhang et al., 2015). It is found that mechanical stiffness and TGF-β can synergistically upregulate the deposition of collagen as well (Figure 2). Transformed myofibroblasts exhibit increased secretion ability of ECM proteins, which further stiffen cardiac tissue and activate fibroblasts, ultimately leading to long-term cardiac fibrosis (Boyle et al., 2021).

#### 2.1.2 Abnormal microarchitecture

ECM is a common meshwork structure that is an important organizer of cell microenvironment. Structural features of the ECM can have profound effects on cell behaviors, which are closely related to the performances and functions of tissues. Cardiac ECM mainly consists of fibrillar collagen, fibronectin, glycosaminoglycans, and proteoglycans, which together provide a stabilized structure and viscoelasticity for cells. Pore size and density of cardiac ECM structure determine the available space and provide a physically confined microenvironment for cell growth (Huang et al., 2017; Frangogiannis, 2019b). Following heart injury, changes in the porosity and density of cardiac ECM will accelerate and regulate inflammation, repair, fibrosis and regeneration. Mainly, caused by the abnormal changes of collagen, the structural remodeling of cardiac fibrosis can affect the forces generated by cells and induce electromechanical transduction processes (Garoffolo and Pesce, 2019; Urbanczyk et al., 2020). The biomechanical properties of fibrotic myocardium are influenced by changes in the quality of collagen fibers, such as the shift in collagen types proportion, increased fibronectin polymerization and increased degree of collagen cross-linking (González et al., 2019). In addition, increased infarct stiffness can prevent left ventricle over-stretch by reducing collagen degradation and facilitating collagen assembly and cross-linking through preservation of the fibronectin network and activation of lysyl oxidase (Voorhees et al., 2015).

The ECM architecture can be rapidly and profoundly regulated by cross-linking reactions enzymatically or non-enzymatically, which can further alter cellular responses and drive disease progression. Collagen cross-linking is a main factor that influences collagen deposition and insolubility of ECM architecture (Neff and Bradshaw, 2021). Firstly, an enzymatically collagen crosslinking that catalyzed by Lysyl oxidase-like 2 (Loxl2) is essential for cardiac fibrosis and mechanical dysfunction of pathological site (Yang et al., 2016). In addition, excess synthesis and activation of the enzyme Lox significantly increases collagen resistance to the degradation by matrix metalloproteinases (MMPs) (El Hajj et al., 2018). Secondly, the imbalance between MMPs and their tissue inhibitors (TIMP) have important effect on the progress of cardiac fibrosis (Polyakova et al., 2011). Thirdly, growing evidence have indicated that transglutaminases (TGs) is involved in molecular responses underlying the pathogenesis of cardiac fibrosis including collagen cross-linking (Al-U'datt et al., 2022). For non-enzymatical collagen cross-linker, proteoglycan fibromodulin has anti-fibrotic effects through regulating collagen fibrillogenesis in cardiac fibroblasts (Andenæs et al., 2018). For example, Kalamajski et al. have documented a fibromodulin-modulated collagen cross-linking mechanism where fibromodulin binds to a specific part of the collagen domain and also forms a complexus with Lox (Kalamajski et al., 2016). Specifically, increasing collagen I:III ratio would provide additional rigidity to tissue structure, whereas decreasing this ratio would provide elasticity and flexibility to the tissue (Kisling et al., 2019). Furthermore, excessive collagen deposition will lead to ECM stiffening, while such stiffening is considered as a pathological change in cardiac fibrosis, which also impairs the compliance of ECM structure, finally altering cardiac tissue microarchitecture (Davis and Molkentin, 2014; Frangogiannis, 2017). Abnormal microarchitecture can affect signal transduction of cardiac cells, promoting cardiac fibrosis through altering the expression of alpha-smooth muscle actin (α-SMA) in cardiac cells (Kuehlmann et al., 2020; Dooling et al., 2022a). Similarly, the abnormal cross-linking of collagen fibers can inhibit the normal renewal of collagen and further promotes the progress of fibrosis.

### 2.2 Transduction mechanism of static biomechanical traits

Cell microenvironment regulates cellular mechanical responses by providing reaction forces to balance intracellular traction. The change of cell microenvironment will lead to the change of association mode and binding strength between cell-cell and cell-ECM, and thus change the cell function and phenotype (Figure 2) (D'Urso and Kurniawan, 2020). The increase in mechanical stiffness and the changes in ECM microarchitecture may affect cell behavior through receptor and signal transduction systems. With the changes in those two static properties, the conformation of structural proteins on the cell membrane becomes unstable, which will activate the signal transduction of biochemical factors and create conditions for changing the gene expression in cells. Increased stiffness and abnormal microarchitecture promote latent TGF-β captured by integrin on myofibroblast membranes and then activate TGF-β (Wipff et al., 2007). In addition, increased stiffness and changed microarchitecture also promote the activity of TGF-β receptor, which is a vital component of the TGF-β/SMADs signaling pathway. TGF-β can promote myofibroblasts to produce ECM proteins and collagen (Leask and Abraham, 2004; Vivar et al., 2013), especially in the process of pathological fibrosis after myocardial injury, which increase the stiffness of damaged myocardium.

Moreover, transient receptor potential (TRP) channels enable cells to convert mechanical signals into chemical signals to accommodate the microarchitecture of the fibrous collagen matrix through myosin contractility during myocardial remodeling (Ji and McCulloch, 2021; Jia et al., 2021). Components of the cardiac ECM can actively communicate with cells as well as the nucleus by binding to cell surface receptors (Kalukula et al., 2022; Miroshnikova and Wickström, 2022). For instance, when ECM is stiffened, active TRPV4 will promote the accumulation of Ca^2+^ in cardiac cells (Van den Bergh et al., 2019). The accumulation of Ca^2+^ can further affect Hippo-YAP signaling to promote cardiac fibrosis (Fukui et al., 2021), for example, promoting cytoskeletal tension and nuclear translocation of Yes-related proteins (YAP) in fibroblast cytoplasm exacerbates adverse cardiac remodeling and impairs cardiac function (Alsamman et al., 2020; Mia et al., 2021). Another factor involved in microarchitectural signal transduction is the myocardia related transcription factor, which relates mechanical stress to the transcription activity of α-SMA genes in various myofibroblast progenitor cells through the polymerization state of actin. Myocardia related transcription factor translocases to the nucleus in a RhoA/ROCK-dependent manner on the rigid substrate, accelerating the fibrosis process (Johnson et al., 2014). Integrin and mature focal adhesions are considered as the main molecular bonds between cells and the ECM microarchitecture, which transmit stiffness and para-tensile signals between cells and their microenvironment (Kechagia et al., 2019).

Huang et al. have found that matrix stiffness-induced cardiac myofibroblast differentiation can be mediated by angiotensin II type 1 receptor (AT_1_R) and Smad7 (Yong et al., 2016). Niu et al. (2020) have revealed that the YAP pathway is a vital signaling branch downstream of AT_1_R receptor in the mechanotransduction of cardiac fibroblast, which may benefit for the development of new treatment of fibrotic diseases. Moreover, Niu et al. (2022) have also discovered a mechanical positive feedback loop between integrin β1 and Piezo1 activation which is initiated by perturbations in matrix stiffness, finally caused the further stiffened environment by fibroblasts. A downstream mediator of mineralocorticoid receptors and insulin receptor activation, the endothelial cell Na^+^ channel (EnNaC), has recently been identified as a key molecular during cardiovascular fibrosis and tissue stiffening (Kleyman et al., 2018). Increased activity of EnNaC results in a number of negative consequences, including stiffening of the cortical actin cytoskeleton in endothelial cells, impaired endothelial NO release, increased oxidative stress-meditated NO destruction, increased vascular permeability, and stimulation of the inflammatory environment (Hill et al., 2022). Furthermore, Liu et al. (2017) have revealed that NIP3-like protein X (BNIP3L) is a novel mediator of ECM structure pressure through the [Ca^2+^]_i_-TGF-β-Smad2/3 pathway in cardiac fibroblasts.

### 2.3 *In vitro* engineering models to simulate static biomechanical traits

Matrix stiffness is considered to be a key static trait that affects not only the physiological development of the heart, but also the pathological state of cardiac fibrosis. Microarchitecture cue is a vital static trait in cardiac fibrosis as well. At present, many researchers have rebuilt various models mimicking those static traits like stiffness or microarchitecture in two-dimension (2D) or three-dimension (3D) to study the specific mechanism during the progression of cardiac fibrosis. These models can provide strong support for the studies of prevention and treatment of cardiac fibrosis (Kong et al., 2019; Jia et al., 2021).

#### 2.3.1 2D models

The 2D cardiac fibrosis *in vitro* models are widely used to study the mechanism of cardiac fibrosis, as well as drug screening. Recently, the role of matrix stiffness in inducing myofibroblast activation can be studied by culturing cardiac fibroblasts with mechanically adjustable gelatin hydrogels (Zhou et al., 2019). For example, Lang et al. (2021) have constructed an *in vitro* cardiac fibrosis model by culturing cardiac fibroblasts on polyacrylamide gels with tunable stiffness to investigate the effect of substrate stiffness on the redox state of cardiac fibroblasts. To determine whether YAP is a modulator of perceived microenvironmental stiffness, Niu et al. (2020) made gelatin hydrogels with different stiffnesses (4–41 kPa) to mimic the stiffness of normal and infarcted cardiac tissue. Under the stimulation of different matrix stiffness, they characterized the ratio of nuclear YAP to cytoplasmic YAP and the expression of total YAP in cardiac fibroblasts (Niu et al., 2020).

Compared to *in vivo* kPa level, the stiffer GPa planar culture dish could not replicate the cardiac tissue stiffness well. The modified planar patterning and modifications create a 2D planar culture environment within controllable, single or multiple factors, morphology, and biomechanical stimulation for cell (Duval et al., 2017; Yang et al., 2021a). To make cell shape controllable and sense static microarchitecture, researchers have created micropatterned matrices in 2D planes to alter the shape of cells (Ma et al., 2017). For example, Yeh et al. (2021) have used decellularized ECM to mimic the native microenvironment and obtained more reliable results that better recapitulate *in vivo* fibrosis. With these models, researchers can focus on the effects of microarchitecture and stiffness perceived by cardiac cells on cardiac fibrosis.

#### 2.3.2 3D models

3D microfluidic technologies have been widely used in all walks of life science, which can provide some new methods to simulate static characteristics of cardiac fibrosis *in vitro* (Marsano et al., 2016; Portillo-Lara et al., 2019). Through precisely positioning biomaterials and living cells in 3D biomimetic models, cardiac fibrosis processes can be simulated using tunable biomechanical models to mimic the diversification of stiffness and structures in cardiac fibrosis. In the 3D scale, researchers developed gels with adjustable stiffness to study the significance of stiffness change in fibrosis. For instance, researchers used tunable and biodegradable hydrogels with different concentrations of the modified HA and methacrylated gelatin to control mechanical stiffness to provide fibroblasts with gradient stiffness stimulations, mimicking the state of cardiac fibrosis *in vitro* (Duan et al., 2013). Mooney et al. have developed a modulated nanoscale architecture to tune the rate of stress relaxation of hydrogels for 3D cell culture to study how the architecture of hydrogels regulates stem cell fate and activity (Chaudhuri et al., 2016). Bhattacharjee and Datta (2019) have prepared a 3D porous architecture media and found that individual cells are intermittently and transiently trapped as they move through the pore spaces. This porous architecture media can provide the picture of bacterial motility in complex media, which can be used to predict cell migration. Bian et al. have reported a supramolecular hydrogel that can provide a controlled platform for investigations on cellular responses to dynamic biophysical cues in 3D environment, and they also found that such hydrogel network has impact on cell behaviors including mechanosensing and differentiation in 3D matrix (Yang et al., 2021b). Sadeghi et al. (2017) developed 3D co-culture *in vitro* model with mixed cardiac fibroblasts and cardiomyocytes in gelatin methacryloyl hydrogel. They proved that the fibroblasts can be kept at rest by imitating the physiology stiffness and cell-cell of natural cardiac tissue. And they have validated the practicability of this model by adding TGF-β1 to activate static cardiac fibroblasts in the model and by analyzing the expression of collagen markers.

Researchers have developed numerous decellularized ECM-based bioinks to construct biomimetic tissue microarchitecture, which provides optimal cell adhesion (Schwan et al., 2016; Jang et al., 2017; Yeh et al., 2021). Wang et al. (2018) developed a functional heart tissue that mimics the microarchitecture, physiological, and functional characteristics of natural heart muscle. This model can be used to regulate the phenotypic transformation of fibroblasts and to study fibrosis structure remodeling. Worke et al. have developed an *in vitro* bionic 3D platform to study cell-ECM interaction, which can help us to understand better how microarchitecture affects chemical signals thus affecting the development and deterioration of cardiac fibrosis (Maji and Lee, 2022). Liu et al. (2020) have studied the shape of fibroblasts by controlling the microarchitecture formed by the 3D hybrid hydrogels. This work showed how cell shape affects the cellular response to 3D mechanical and biochemical cues, and has implications for the development of cell shape modulation-specific approaches to treat fibrosis. Worke et al. (2017) have also explored the potential of compressed collagen matrix as a structure mechano-chemical and physiochemical related cardiac fibrosis model system by combining collagen with embryonic cardiomyocytes. In brief, these models provide us with strong support to study static biomechanics of cardiac fibrosis *in vitro* and test anti-fibrotic drugs in promoting real-time assessment of cardiomyocyte function.

## 3 Dynamic biomechanical traits

### 3.1 Common dynamic biomechanical traits during cardiac fibrosis

#### 3.1.1 Stretch

The heart is a dynamic organ with the ability to contract and relax in a coordinated manner in space and time. On the organ scale, the dynamic stretch caused by the heartbeat is the predominant biomechanical feature of the myocardium. The progression from injury to fibrosis is a rigid process filled with aggressive immune cells and reconstructed sites, and delayed onset of ventricular stretch can be expected. On the cellular scale, the dynamic stretch originates from the beating of cardiomyocytes and partly from the contraction of myofibroblasts. Recent studies have shown that excessive dynamic stretch may be a powerful stimulator for continuous activation of myofibroblasts and remodeling of cardiac ECM, enabling fibrosis to progress (Kong et al., 2019; Walker et al., 2020). The loss of functional tissue and the reduction of myocardial elasticity and contractility lead to a vicious circle of low mechanical efficiency, which finally causes negative fibrotic remodeling, impaired stretch, and ultimately heart failure (Richardson et al., 2015; Long et al., 2022).

The dynamic contraction properties of cardiac tissue have a vitally biomechanical effect on cardiac fibrosis. Perception of external abnormal stretch signals by cardiac cells and the subsequent biomechanics of cell-ECM interactions can regulate downstream mechanotransduction events (Maurer and Lammerding, 2019), e.g., the differentiation of fibroblasts in healthy myocardium into pathological myofibroblasts in response to mechanical stretch overload of cardiomyocytes. Myofibroblasts are distinctive with the presence of a large number of contractile apparatuses containing actin filaments and related proteins (Mia et al., 2021). These contraction devices have a mechanical conversion function which allows myofibroblasts to convert the excessive stretch they perceived into chemical signals. In addition, actin filaments in myofibroblasts can alter cell shape, promote cell movement and transmit forces to the surrounding matrix environment resulting in ECM reorganization and contraction (Sandbo et al., 2016). Specifically, at the development of fibrosis, fibroblasts undergo phenotypic conversion into myofibroblasts by developing muscle-like features, including formation of contractile actin-myosin bundles (Hinz et al., 2019). Actin and myosin filaments work together to generate force to alter cell shape. It has been reported that the actin filaments may make cell shape more polarized. Due to the fibroblasts had long protrusions, the cells in 3D were stellate in shape, with numerous projections, and thus similar in shape to fibroblasts on 2D tissue culture plastic (Kalson et al., 2015; Yeung et al., 2015). Caused by the abnormal stretch, abnormal constriction of cardiac blood vessels increases and accelerates the progression of heart failure during cardiac fibrosis (Wu et al., 2021).

#### 3.1.2 Fluid shear stress

The human cardiac is a marvelous fluidic system, which is very sensitive to biomechanical and biochemical. As a dynamic biomechanical trait, fluid shear stress is mainly derived from blood flow in the heart (Wang et al., 2022). Multiphasic fluid is contained about many parts. Such as, intracellular stress fibers filled with intracellular fluid at the molecule level, a fibrous network filled with tissue gel in the extracellular matrix at the cellular level, blood flow in capillaries at the tissue level (Feng et al., 2014). Among these types of matter, multiphasic fluids in cardiac show an orderly multiscale spatial flow that determines biological activities (Liu et al., 2022; Stine, 2022). Mechanical shear from blood flow has a major impact on endothelial cell physiology and a key role in initiating vascular regulatory signaling. Appropriate shear stress maintains endothelium homeostasis, while abnormal shear stress in the fibrotic microvascular system may elicit endothelial dysfunction. Endothelial cells do not normally experience the fluid shear of blood flow, but are activated by cytokines at sites of the fibrosis (Kreuger and Phillipson, 2016). In addition, shear stress can modulate the mechanical sensitivity of human blood mononuclear cells (Baratchi et al., 2020). Fluid shear changes at abnormal sites during cardiac fibrosis often cause a range of changes, such as affecting cardiac tissue and cellular morphology.

Caused by the abnormal contraction of the myocardium, the abnormal contraction of blood vessels can lead to higher fluid pressure, and causes abnormal fluid shear stress in the vascular endothelium. The shear stress on endothelial cells can be transmitted to other cells *via* cell-cell and cell-ECM interaction. Continuous blood flow shear loading is also thought to contribute to fibroblast proliferation, migration and differentiation. This process leads to fibrosis, which promotes the progression of many cardiac diseases by blocking myocardial excitation-contraction coupling and interfering with pulse propagation and ECM-dependent signaling pathways. Therefore, over-stretch and fluid shear loads regulate the function of many mechanosensitive ion channels and transmembrane proteins, which interact closely with the ECM to activate a range of signaling pathways to alter cellular function to affect the progression of cardiac fibrosis (Priya et al., 2020).

### 3.2 Transduction mechanism of dynamic biomechanical traits

From the biomechanical point of view, myocardial tissue can be regarded as an elastic material. Thus, we can explore the specific biomechanical transduction mechanisms caused by abnormal heart contraction in the process of fibrosis from the aspects of dynamic tension and fluid shear stress (Figure 2) (Dooling et al., 2022b; Villalobos Lizardi et al., 2022). Interestingly, the behavior of cells subjected to over-cyclic stretch on flexible substrate is similar to that on a rigid substrate, which indicates that over-cyclic stretch can replace rigid substrate in stimulating fibroblasts spreading, stress fiber formation and growth (Qian et al., 2013; Cui et al., 2015). The expression of α-SMA can make cells produce mechanical stress, which not only plays an important role in tissue reconstruction and contraction, but also can be used as a mechanical transducer to connect mechanical sensing factors and increase their expression in stretch induction (Balachandran et al., 2011; Zhao et al., 2022b). Myofibroblast contraction activates latent TGF-β from the ECM, which can promote fibroblasts to differentiate into myofibroblasts through inducing smooth muscle myosin and α-SMA expression (Walker et al., 2020). The dynamic contraction of matrix architecture can promote the activation of TGF-β as well, which adds new content to the complex “indirect mechanical induction” mechanism (Hanna et al., 2021). For example, besides promoting cell attachment to microstructure through adhesive spots, TGF-β can also facilitate the specific binding of integrin to the hidden domain (exposed under cyclic stretching) of fibronectin ligands (Zarubova et al., 2022).

Moreover, mechanical sensitive channels on the cell membrane also play an important role during cardiac fibrosis (Saucerman et al., 2019; Stewart and Turner, 2021a). Stretch-activated channels are non-selective cation channels that increase in activity in response to mechanical stress (Lorin et al., 2015). Some stretch-activated channels are thought to contain ion channel proteins from a large family of TRP channels, such as TRPV2 or TRPV4. In the vascular endothelium, local Ca^2+^ influx through TRPV4 plays a vital role in endothelial cell adaptation to hemodynamics (Baratchi et al., 2017). Mechanosensitive Ca^2+^ permeable ion channels are an important class of proteins expressed on circulatory blood cells, responding to mechanical stimuli and participating in the sensing of shear stress (Baratchi et al., 2020). The increase in stretch and fluid shear force caused by cyclic strain can promote the deformation of piezo-type mechanosensitive ion channel component 1 (Piezo1), which in turn promotes cytoskeletal remodeling (Chowdhury et al., 2021; Lai et al., 2021; Liao et al., 2021). In addition, fluid shear stress can also induce TGF-β and angiotensin Ⅱ type 1 receptor (AT_1_R) signals, thus promoting cardiac fibrosis (Stewart and Turner, 2021b; Yang et al., 2021c).

Calcium signaling is also fundamental to cardiac ECM microarchitectural contractility (Terrar, 2020). Due to the regular contraction and relaxation of the myocardium, both fibroblasts and myofibroblasts are affected by cyclic strain in both flexible and rigid microarchitecture. Researchers have found that RhoA is involved in regulating calcium release in response to cardiac stress (Lauriol et al., 2014; Mathiyalagan et al., 2019; Dridi et al., 2020). Studies have shown that dynamic mechanical stimulation can facilitate the release of the PDGF and Angiotensin II (Ang II) (Lisy et al., 2000; Lopez-Bellido et al., 2019). Over expression of PDGF and its receptors, including PDGFR-α and PDGFR-β, can lead to cardiac fibrosis and cardiac ECM protein deposition (Kong et al., 2014). Ang II, a key mediator of the renin-angiotensin-aldosterone system involved in cardiac remodeling (Schorb et al., 1993; Jong et al., 2016), is usually elevated after myocardial injury and causes cardiac fibroblast proliferation and collagen overexpression (Cai et al., 2019; Jana et al., 2021). However, the effect of cardiac ECM microarchitecture on Ang II-induced cardiac remodeling and heart failure remains unknown (Chen et al., 2021). Developing the methods of dynamic biomechanical traits and cardiac cells may find a new therapeutic target for fibrotic diseases.

Cadherin-11 has been described as a senescence-responsive molecular, its expression is suppressed in senescent endothelial cells and such suppression is greater when senescent cells are under shear stress (Mun and Boo, 2010). In addition, cilium of endothelial cells is abundant in regions subjected to low shear stress or disturbed blood flow, while absent in regions with high shear stress (Garoffolo and Pesce, 2019). Furthermore, mechanical deformation of focal adhesion proteins would elicit the activation of stretch-dependent signaling pathways (Riley and Merryman, 2021). For example, focal adhesion kinase is activated by cyclic stretch, which then activates the protein kinase B (AKT) and mitogen-activated protein kinases (MAPK) pathways to promote myofibroblast differentiation (Zebda et al., 2012).

### 3.3 *In vitro* engineering models to simulate dynamic biomechanical traits

Establishment of *in vitro* models in 2D or 3D allows for the integration of other relevant biomechanical stimuli important to the human myocardium, such as cyclic stretch and ECM structures (Rogers et al., 2019; Veldhuizen et al., 2020). Cardiac fibrosis biomechanical models have attracted extensive attention due to their inclusion of vital dynamic traits. By simulating dynamic stimulus on classic 2D models or stereoscopic 3D models (Bein et al., 2018; Yu and Choudhury, 2019; Vivas et al., 2022), we can mimic dynamic biomechanical traits of cardiac fibrosis *in vitro* well at the multiscale (Table 1).

**TABLE 1:** Examples of modeling biomechanical traits in 2D or 3D cell culture.

Models	Biomechanical traits	Uses	Refs
2D			
Oscillating shear model	Fluid shear stress	The activation method of TGF-β1 ubiquitously in a latent form	Kouzbari et al. (2019)
Mechanochemical modeling framework	Cyclic stretch and sub-cellular structures	Predicting the preferred alignment of cells under stretch	Qian et al. (2013)
Hydrogel-based system	Simulation of matrix stiffness after myocardial tissue infarction	Modulating myofibroblast mechanotransduction	Zhao et al. (2014)
Photodegradable PEG based hydrogel system	Stiffness and structure	Spatially varying matrix elasticity and studying the effect of matrix elasticity organization on valvular interstitial cells phenotype	Ma et al. (2017)
Gelatin hydrogels platform	Stiffnesses (4 and 36 kPa)	The proliferative ability of the cardiac fibroblasts cultured on substrates with different stiffnesses	Niu et al. (2020)
Decellularized ECM experimental platform	Microarchitecture and stiffness	Mimicking the native microenvironment more accurately	Yeh et al. (2021)
Engineered biohybrid constructs	Myocardium laminar structure	Measuring myocardial contractility	Grosberg et al. (2011)
3D			
The micro-physiological system	Cyclic stretch	Up to six different biologically independent samples are incorporated in a single device	Mainardi et al. (2021)
3D microscale cell-laden hydrogel platform	Cyclic stretch (10% strain at 1 Hz)	The recapitulation of key stages of cardiac fibrosis (i) proliferation, (ii) fibroblast to myofibroblast phenotypic switch, (iii) matrix deposition and (iv) stiffening	Occhetta et al. (2018)
3D model of human cardiac fibrosis	Stretch triggered by human induced pluripotent stem cell derived cardiomyocytes	The classic hallmarks of fibrosis-induced heart failure including high collagen deposition, increased tissue stiffness, BNP secretion, and passive tension	Mastikhina et al. (2020)
Laser-cut sheets of decellularized myocardium scaffolds	Cyclic stretch, shear stress and microstructure	Assessing the nature of the organization	Jang et al. (2017)
The MVAS-force model	Stretch and microstructure	Linking cell-level phenotypic changes to functional changes	Walker et al. (2020)
The beating heart-on-chip device	Cyclic stretch and microstructure	The beneficial effect of mechanical stimulation on the functional maturation of cardiac microtissues	Ugolini et al. (2018)
3D GelMA-based hydrogel platform	Stretch and stiffness	The activation of cardiac fibroblasts into myofibroblast	Sadeghi et al. (2017)
3D microfluidic cardiac tissue model	Tissue architecture	Recapitulating the native myocardium	Veldhuizen et al. (2020)

#### 3.3.1 2D models

**FIGURE 3:**
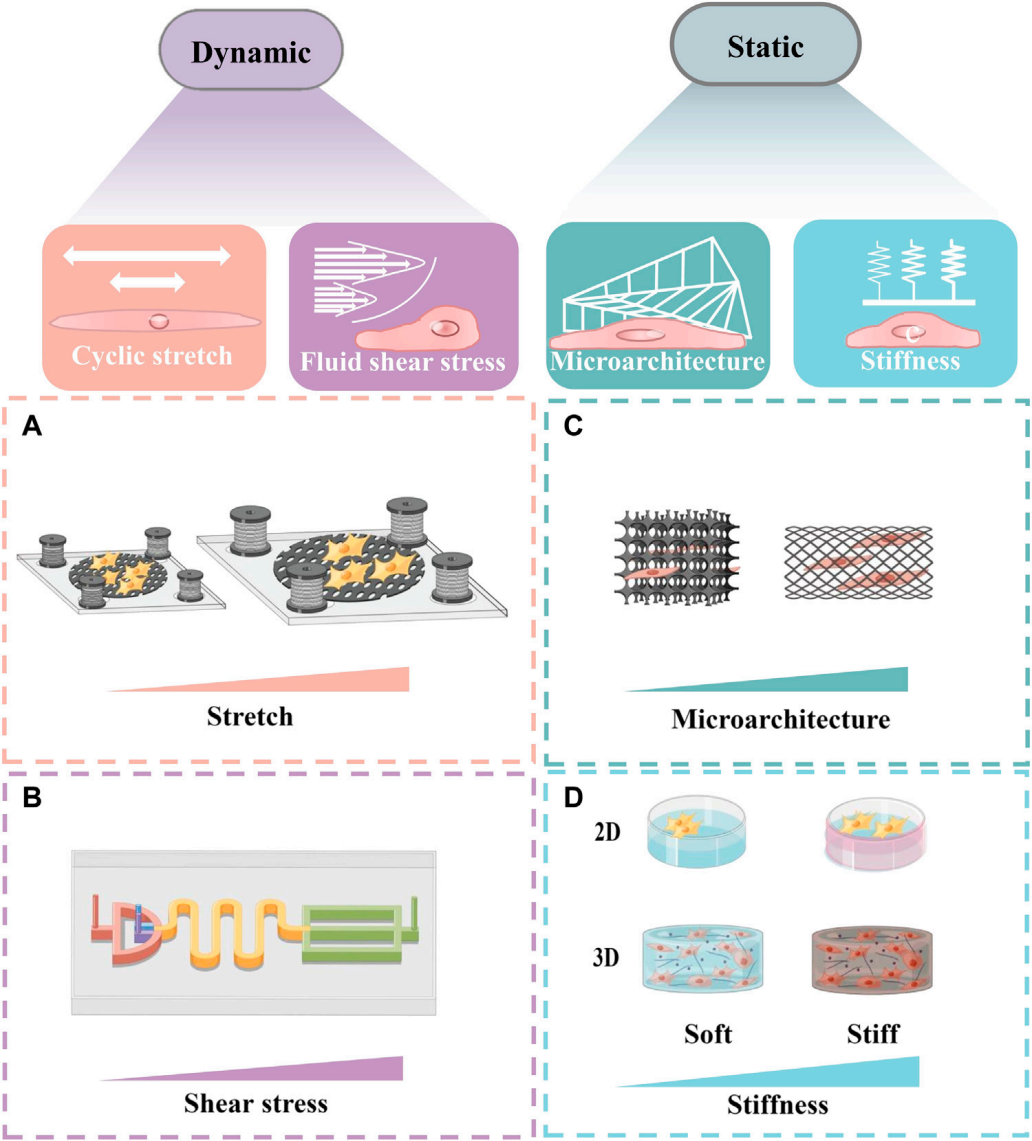
Schematic of several in vitro models simulating the change of biomechanical traits. Schematic diagrams were drew online using Figdraw. **(A)** A schematic diagram of a stretch system to apply long-term cyclic stretch to cells. **(B)** A schematic diagram of the equipment, which can generate shear force and rotational shear force respectively to simulate the fluid shear stress *in vitro*. **(C)** A scalable microarchitecture-cultivation platform for engineering cardiac tissues. **(D)** A schematic diagram of cells cultured in different matrix stiffness in 2D and 3D.

Researchers have performed cell culture experiments on various stretchable 2D planes to simulate abnormal stretching that appears in cardiac fibrosis. For example, stretching the cellular structure can recapitulate part of the complex mechanical environment that cardiovascular cells experience *in vivo* (Figure 3A) (Cirka et al., 2016). Studies in which dynamic mechanical stress was applied to cardiac fibroblasts by stretching silicon films revealed a significant increase in fibrotic responses, including cardiac fibrosis, fibroblast proliferation, collagen expression and matrix metalloproteinase *in vitro* (Yuan et al., 2018). Grosberg et al. (2011) implanted cardiomyocytes on the surface of elastic films to form a layer of cell sheet, such films would deform regularly with the beating of cardiomyocytes. The chip can not only monitor the contractility and electrophysiological properties of cardiomyocytes in real time, but also can be used in pharmacological studies.

Microfabrication can generate shear and rotational shear forces to simulate fluid shear forces (Figure 3B) (Kouzbari et al., 2019). Such technology can provide us with a method to simulate the abnormal fluid shear forces generated in cardiac fibrosis. Chester et al. (2018) used microgel films with viscoelastic properties to explore the significant effect of material stretch on cell adhesion, migration, or myofibroblast differentiation. They found that stretch stimulation had a facilitative effect on early fibrotic response.

#### 3.3.2 3D models

The construction of the 3D cardiac fibrosis pathological model *in vitro* is of great significance for the study of dynamic traits in cardiac fibrosis. For instance, through controlling the concentration of collagen quantitatively and the density of fibroblasts in the cardiac tissue models, van Spreeuwel et al. (2017) found that the increase in the number of fibroblasts significantly reduced myocardial contractility and altered the heart rate. Kong et al. (2019) studied the relationship between mechanical movement and cardiac fibroblast proliferation by applying cyclic compression of gradient amplitude and adjustable frequency on GelMA hydrogel containing cardiac fibroblast. They found an important correlation between stretch stimulation and phenotypic transformation of cardiac fibroblast, which provides new ideas for the prevention and treatment of cardiac fibrosis in the future.

At present, a variety of “cardiac tissue chips” have been developed to simulate and manipulate dynamic mechanical microenvironment of cardiac tissue at the micro-scale by combining with microfabrication or microfluidic technology, providing real-time insights into fibrosis events. Moreover, cardiac tissue chips can offer an extraordinary way to precisely manage different microenvironment signals (e.g., abnormal stretch or fluid shear stress) to construct biomimetic 3D *in vitro* cardiac fibrosis models (Duval et al., 2017; Soon et al., 2021). For example, Zhao et al. (2019) described a scalable tissue-cultivation platform that is cell source agnostic and enables drug testing under electrical pacing (Figure 3C). This controlled 3D platform with multiple cells enabled real-time recordings of cardiomyocytes’ active tension, passive shear force and dynamic stretch. Mainardi et al. (2021) developed an *in vitro* fibrosis model which could help to uncover new pathological aspects and to study the crosstalk between cyclic stretch and the most abundant cell types involved in fibrosis.

Additionally, 3D *in vitro* cardiac fibrosis models can better simulate natural myocardial tissue *in vivo*, and also can better mimic complex interactions of cell-cell and cell-ECM, complex mechanical traits and chemical signals (Figure 3D) (Marsano et al., 2016). Occhetta et al. (2018) constructed a 3D *in vitro* cardiac fibrosis model by cyclically stretching cardiac fibroblasts embedded in a 3D hydrogel to simulate an *in vitro* fibrosis-like microenvironment. They have reproduced some of the major fibrosis features within 7 days, and cyclic strain did increase fibroblast proliferation and ECM deposition, resulting in a higher quality scar-like tissue. 3D dynamic devices *in vitro* have provided futuristic platforms for elucidating cardiac ECM remodeling, fibrosis pathophysiology and dynamic contractile function (Savoji et al., 2019), which may provide direction for exploring the therapeutic targets for cardiac fibrosis.

## 4 Conclusion and outlook

Besides those talked about above, other biomechanical properties in cardiac fibrosis also deserve our attention, like the viscoelastic and anisotropic of the cardiac tissues. Here we have only focused on the elastic enhancement of cardiac fibrosis (increased stiffness) and ignored the changes in viscosity, which adversely affect the understanding of the mechanism of cardiac fibrosis. Noteworthy, for the whole heart, the myocardial tissue is arranged in a spiral shape relative to the ventricular axis, and the forces on cells in different parts of the ventricular wall are anisotropic. The biomechanical behaviors of cardiac tissues at the macroscale are tightly coupled with cellular activities at the microscale, such as cardiac cell perception of external physical signals and subsequent dynamic regulation of cell-ECM interactions downstream of mechanotransduction events (Guo et al., 2022; Zuela-Sopilniak and Lammerding, 2022). Moreover, the connection between the whole cardiac mechanics and the mechanical traits of the fibrotic sites was not mentioned. A comprehensive understanding of the biomechanical features of cardiac fibrosis requires a rigorous and broad perspective on biomechanics and fibrosis. Future research should detail the biomechanical traits of cardiac fibrosis (Park et al., 2022), and more in-depth studies and explorations are needed.

Despite the remarkable progress so far, the comprehensive mechano-regulatory mechanisms of cardiac fibrosis remain elusive, which creates a great need to study cardiac fibrosis further. And finding effective anti-fibrosis therapy will make a significant contribution to the treatment of cardiac fibrosis as well as countless other fibrotic diseases. Because the cardiovascular system is constantly subjected to mechanical forces, increasing researchers have been taking the impact of changes in cardiac biomechanics on cardiac fibrosis seriously (Lyon et al., 2015). Cardiac fibrosis models *in vitro* simulating the process of fibrosis have been a crucial support for the study of effective anti-fibrosis therapy (Deddens et al., 2017), simulating these biomechanical traits *in vitro* may help to explore specific drugs for treating fibrosis. For example, Zhao et al. (2014) fabricated patterned hydrogels using photolithography to simulate the different stiffness of the ECM and the characteristics of matrix architecture, which can be used to test the anti-fibrotic efficacy of candidate drugs and has potential in the study of fibrosis pathology. Rogers et al. (2019) incorporated cardiomyocytes and fibroblasts into a fibrin gel to construct a 3D tissue in a cardiac tissue chip (which undergoes cycles of stretch, pressure, ejection, and relaxation similar to those observed during the cardiac cycle), to explore new drugs to treat cardiac fibrosis. Some investigators simultaneously controlled a variety of complex factors such as chemical cues, mechanical force stimulation, and biological fluids, thereby simulating the structure and functional characteristics of cardiac tissues. Many current models have been able to revolutionize biomedical applications by better mimicking natural tissues (Duval et al., 2017; Rodríguez‐Cabello et al., 2022). With these efficient and simulated biomechanical models, these *in vitro* models have broad prospects in studying the physiological and pathological mechanisms of cardiac fibrosis, as well as screening pro-fibrotic and anti-fibrotic drugs. Maybe we can have deeper research on the physiology and pathology of cardiac fibrosis and other diseases in the human body in the future.

Understanding the origin and consequences of the biomechanical characteristics of cardiac fibrosis is a key principle that is critical to improving treatment. Many of the concepts involved are non-intuitive and require a deep and broad understanding of the characteristics and biomechanics of fibrosis. In conclusion, the complexity of biomechanics in the cardiac fibrosis microenvironments requires further exploration using groundbreaking technologies for the exquisite recapitulation of mechanical crosstalk during fibrosis progression and prediction. We believe understanding these dynamic and static biomechanical traits should pave the way for effective anti-fibrosis strategies for clinical therapy.
